# Psychosis in a male due to Coronophobia-, psychological impact of COVID-19 pandemic in India

**DOI:** 10.1192/j.eurpsy.2023.1705

**Published:** 2023-07-19

**Authors:** R. Tripathi, M. Prithviraj

**Affiliations:** All India Institute of Medical Sciences, Gorakhpur, India

## Abstract

**Introduction:**

Change in any form is threatening and so is the change due to COVID-19 infection. In the second wave of COVID-19 pandemic in India, many have been infected with coronavirus and many have lost their lives. There was a surge of anxiety, depression and suicide. The impact on psychological functioning also has been immense. There has been a surge in anxiety and depression as the major tool typically used to cope with stress, such as social support, couldn’t be utilized properly. The fear of acquiring COVID 19 infection (coronophobia) and using excessive hygiene measures were also on the rise (3,4). The fear has become more pronounced as living with coronavirus with constant precautions has become the new norm

**Objectives:**

We would like to present a case report in which the patient developed psychosis due to fear of acquiring COVID 19 infection

**Methods:**

Case-report

**Results:**

The first patient was a 37 years old male, farmer who has onset of his symptoms during Covid-19 pandemic in India in 2020. He would be restless and fearful all the time and would take necessary precautions and follow all the necessary hygiene protocols. During the first wave of COVID-19 pandemic in India, there were few deaths in his locality. This made him more restless and fearful and he began to believe that he also had acquired covid infection. Despite repeated negative results for SARS-Cov 2, he would deny the results findings. He developed psychotic symptoms during second wave of the COVID-19 pandemic. He was managed on antipsychotics with full remission in six months.

**Conclusions:**

A great deal of attention should be paid to the diagnosis, course and treatment of anxiety caused by COVID-19. If left untreated, it could trigger greater problems such as psychosis as in our case

**Disclosure of Interest:**

None Declared

